# Three-axis MR-conditional robot for high-intensity focused ultrasound for treating prostate diseases transrectally

**DOI:** 10.1186/s40349-014-0023-2

**Published:** 2015-01-29

**Authors:** Christos Yiallouras, Kleanthis Ioannides, Tetiana Dadakova, Matt Pavlina, Michael Bock, Christakis Damianou

**Affiliations:** MEDSONIC LTD, Limassol, Cyprus; Electrical Engineering Department, Cyprus University of Technology, Limassol, Cyprus; Biomedical Engineering Department, City University, London, UK; Radiology, Polikliniki Igia, Limassol, Cyprus; Radiology—Medical Physics, University Medical Center Freiburg, Freiburg, Germany

## Abstract

**Background:**

A prototype magnetic resonance image (MRI)-conditional robot was developed for navigating a high-intensity focused ultrasound (HIFU) system in order to treat prostate cancer transrectally.

**Materials and methods:**

The developed robotic device utilizes three PC-controlled axes: a linear axis for motion along the rectum, an angular axis for rotation in the rectum, and a linear axis to lift the robot up and down. Experiments with the system were performed in a 1.5-T MRI system using gel phantoms.

**Result:**

The robot was successfully operated in a 1.5-T clinical MRI system. The effect of piezoelectric motors and optical encoders was quantified based on the reduction of signal to noise ratio. Discrete and overlapping lesions were created accurately by moving the HIFU transducer with the robotic device.

**Conclusion:**

An MRI-conditional HIFU robot was developed which can create controlled thermal lesions under MRI guidance. The intention is to use this robot transrectally in the future for the treatment of prostate cancer.

## Introduction

The idea of using magnetic resonance imaging (MRI) to guide and monitor high-intensity focused ultrasound (HIFU) was historically cited for the first time in 1992 by Hynynen [1], and at that time, it was shown that thermal lesions created by HIFU on canine muscle *in vivo* can be clearly seen using MRI.

The MRI-guided HIFU robot produced in the subsequent years [2] used hydraulic principles to move the transducer. However, this hydraulic robot had serious problems with accuracy due to the accumulation of bubbles caused by water leaks coming from the compression of water in the tubes.

The next generation of HIFU positioning devices made use of piezoelectric motors which are MRI-conditional. The piezoelectric motors in HIFU were introduced by the Israeli company InSightec [3]. This technology resulted in the first commercial system for the treatment of uterine fibroids, which received approval by the Food and Drug Administration (FDA) in 2004. This system is incorporated in the table of a General Electric MRI scanner. The system initially was approved for the treatment of various gynecological tumors [4-9]. An endorectal system produced by the same company has been utilized recently for the treatment of prostate cancer [10,11]. A body system is currently used by InSightec for pain palliation of bone metastases [12-14]. A transcranial system is currently used by InSightec for non-invasive treatment of various brain diseases [15] such as brain cancer, stroke, and Parkinson disease.

Philips Healthcare, Netherland, showed interest in this technology recently, and as a result, the MRI-guided HIFU system Sonalleve was developed as a commercial product [16] for the treatment of uterine fibroids and bone palliation. This system is incorporated in the table of a Philips MRI scanner.

The role of HIFU for the treatment of benign prostate hyperplasia (BPH) appeared in the 1990s [17,18]. In those studies, HIFU was delivered via a transrectal probe with ultrasound imaging capabilities.

HIFU was utilized on prostate carcinoma in rats, where it was proven that HIFU has the potential to treat small localized prostate cancer lesions [18]. Clinical trials utilizing HIFU and ultrasound imaging were published thereafter [19,20]. The two dominant clinically available systems using transrectal HIFU are Ablatherm HIFU (Technomed International, Lyon, France) and HIFU Sonablate 500 (Focal Surgery, Milpitas, CA now SonaCare Medical, Charlotte, North Carolina). Recent advances of the transrectal device include a phased array probe for more efficient treatment of prostate [21].

Chopra et al. developed a prototype system for MRI-guided transurethral HIFU therapy [22,23]. The transurethral HIFU probe includes a planar transducer and is capable of heating locally around the urethra, thus delivering controlled thermal therapy to the prostate gland.

The development of MRI-guided HIFU was inspired by the development of MRI-compatible positioning devices for other interventional methods. Stoianovici et al. [24] were the first group of researchers to develop a pneumatically actuated MRI-save compatible robot designed for transperineal interventions in the prostate. In the study reported by Zangos et al. [25], an MR-compatible transrectal prostate biopsy actuator was developed. The actuator uses pneumatic motors. Pressured air in these motors is generated in the controller unit and is transmitted through the plastic hoses. With five degrees of freedom (DOF), the needle guide can be manipulated in the desired position. Another example of interventional device is the MRI-compatible robot for prostate brachytherapy [26].

Real-time MR temperature mapping provides essential information about the location of the focal spot, but the well-defined and rapid movement of the focus requires either the use of a phased array US transducer with many US elements to electronically steer the US beam or a mechanical robotic device to mechanically move the fixed focus of a US transducer with only very few US elements. In this work, a dedicated mechanical device is designed to move and position a transrectal US transducer during an MR-guided HIFU procedure at a clinical field strength.

The proposed robotic system is an extension of the robotic system for brain ablation [27] which includes three Cartesian axes. The proposed system includes two linear stages and one angular stage. The system has been inspired by the existing HIFU systems with ultrasonic imaging [17,18]; however, it uses safe materials to be able to perform MRI imaging for guidance and monitoring. The robotic system is designed to be applied transrectally. Our robotic system uses only MRI-safe or MRI-conditional materials such as piezoelectric ultrasonic motors (Shinsei USR60E3N), optical encoders, and acrylonitrile butadiene styrene (ABS) plastic. Two PC-controlled axes are needed for linear motion along the axis of the rectum (*X*) to position the transducer over the lesion in the prostate gland and in up-down direction (*Z*) adjusting the system to the variable height of the prostate relative to the MRI table. The angular axis (*θ*) is used to rotate the transducer around the *X*-axis (i.e., the rectum) so that different angular positions in the prostate can be treated.

The robotic system is manufactured using a digital manufacturing device (FDM400, Stratasys, 7665 Commerce Way, Eden Prairie, Minnesota, 55344, USA).

The MRI compatibility of the system was evaluated in a clinical MRI scanner. The robot was evaluated in creating thermal lesions in commercial gel phantoms. The robot was tested for its ability to accurately move the transducer, thus creating discrete and overlapping thermal lesions. The imaging of the lesions was performed using the optimized techniques described in [28] and [29].

## Materials and methods

### Robotic system

- Mechanical design of the positioning device.

The entire robotic system was developed using the software MicroStation (V8, Bentley Systems, Inc.). After completion of the design, drawings of the individual parts were sent to a 3D printer (FDM400, Stratasys, 7665 Commerce Way, Eden Prairie, Minnesota, 55344, USA) for production. This 3D printer produces parts made out of ABS.

The three axes were driven by piezoelectric ultrasonic motors (USR60-S3N, Shinsei Kogyo Corp., Tokyo, Japan). The positioning device was designed so that it can be placed on the patient table of any conventional high-field MRI system with a cylindrical magnet bore. It has a maximum height of 17 cm, a length of 25 cm, and a width 20 cm which allows placing the device between the legs of the patient during treatment in the MR system. Here, it was assumed that the patient is lying in prone position on the table with slightly elevated legs to provide an optimal access to the rectum for the US transducer. The motion range of the robot was set to *X*: 10 cm, *Θ*: ±90°, *Z*: 7 cm, which is sufficient to treat prostate glands up to a size of 10 cm length. The robotic system weights around 2 kg.

The positioning device has the ability to move the transducer linearly up to 10 cm. Additionally, the transducer can be moved at an angle of ±90°. The linear range of this prototype covers the size of all possible prostates. It is possible, that in the future, the linear range might be decreased, thus reducing the size and cost of this device. The angular range, which moves clockwise or counterclockwise by 90°, covers any potential discrete tumor in the prostate.

**Figure 1:**
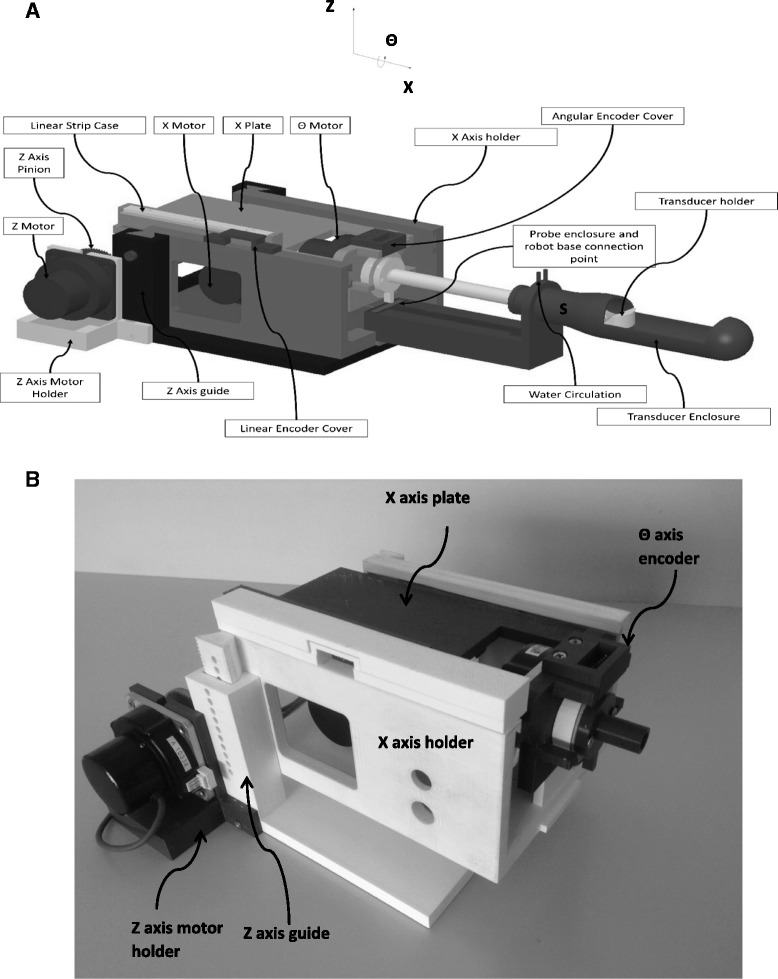
**Complete CAD drawing and photo of the positioning device. (A)** Complete CAD drawing of the positioning device, and **(B)** photo of the positioning device with the three PC-controlled stages.

Figure 1A shows the complete CAD drawing of the robotic system. Note that the *X*-axis as described in the robot drawing is the *Z*-axis in MRI, and the *Z*-axis in the drawing is the *Y*-axis in MRI. The main body of the robot (*Z*-guide) moves up or down (*Z*-axis) since the height of the rectum is variable. The *X*-axis holder provides guides for the *X*-plate which establishes linear movement. The *X*-axis holder includes also a motor holder for the *X*-stage and one optical encoder (EM1, US Digital Corporation, Vancouver, WA 98684, USA). The *X*-axis plate includes a motor holder for the *Θ*-axis. In the bottom of the *X*-plate, a rack is created which is attached to the motor in the base which carries a brass pinion (Sterling Instrument, NY, USA). The motor in the front of the *X*-plate establishes angular motion (*Θ* stage). An angular encoder (US Digital Corporation) is placed on the shaft of the motor and ensures accurate angular movement of the shaft. A water enclosure which is attached into the base accommodates two thin water inlets for water cooling of the transducer. The transducer is attached to the shaft of the angular stage. The water enclosure will be inserted in the rectum in future clinical trials. At the far end (point S in Figure 1A), the width of the water container is smaller, because at this point, the sphincter of the patient will surround the water container, and thus, pressure to the sphincter has to be reduced. Figure 1B shows the photo of the positioning device with the three PC-controlled stages.

Three optical encoders were used from US digital (EM1, US Digital Corporation, Vancouver, WA 98684, USA). The EM1 optical encoders work in conjunction with a polymer plastic strip materials, and it uses LED source and a monolithic detector. For the two linear axes, the EM1-0-500-I (US Digital Corporation) encoder was used, whereas for the angular motion, the EM1-2-2500-I (US Digital Corporation) encoder was integrated. The encoder output is connected to the counter input of a data acquisition board USB 6251 (NI, Austin, USA).

Because of the use of piezoelectric motors and encoders that require electricity during operation, the proposed robotic system is classified as MRI-conditional, according to the ASTM standards (F2503, F2052, F2213, F2182, and F2119) which are excellently described by Stoianovici et al. [30].

The motion accuracy of the three motors was evaluated using a digital caliper. One edge of the caliper was fixed on a stationary part of the positioning device, and the other edge was fixed on a movable part. The movement of a specific step was measured using the incremental distance of the caliper. The resolution of the caliper was 10 μm. The accuracy of the robotic system was tested by measuring the distance moved based on a predetermined number of pulses of the encoders.

b) Software

**Figure 2:**
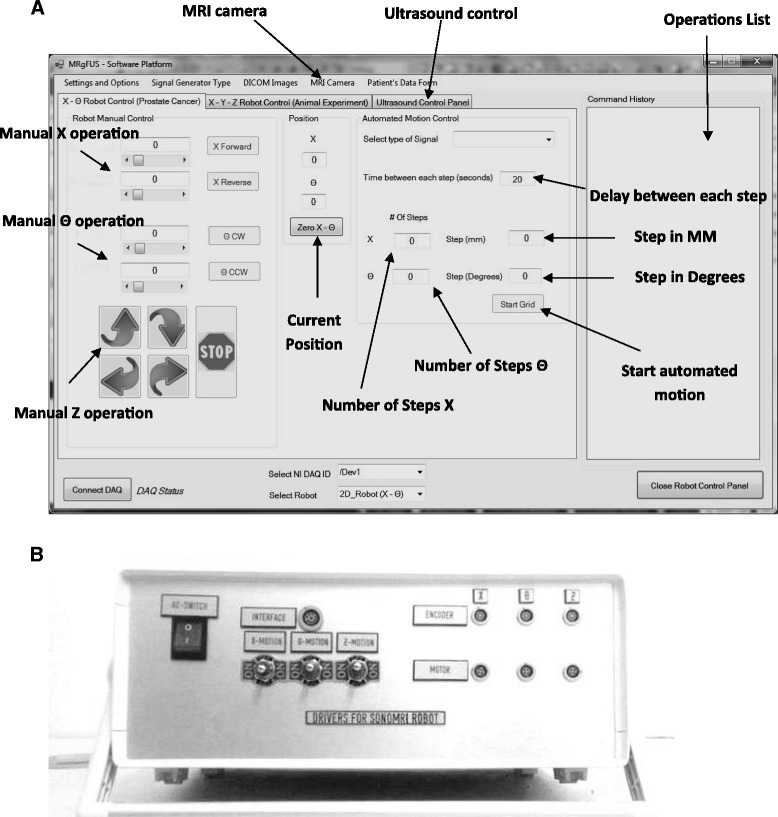
**Main window of the software and electronic system. (A)** Software used to control the positioning device and the therapeutic system which is guided by MRI, and **(B)** electronic system.

A user-friendly program written in C # (Visual Studio 2010 Express, Microsoft Corporation, USA) has been developed in order to control the robotic system. The software has the following functionalities: a) communication with MRI. The communication is achieved by an Ethernet connection and a laptop; b) movement of the three axes either in user defined steps or in grid sequences by specifying the pattern, the step, and the number of steps. The interface used is the USB 6251 data acquisition (DAQ) interface card (National instruments, Austin, Texas, USA) via a connecting block. The USB 6251 interface card includes timing and digital I/O modules; c) history (functions activated); d) patient information; e) transducer coordinates; f) images of an MR-compatible camera (MRC Systems GmbH, Heidelberg, Germany). The camera was interfaced by means of a video card; and g) temperature measurement. An Omega (M2813-1205, OMEGA Engineering, INC. Stamford, Connecticut, USA) voltage to temperature converter was used to measure the temperature. Figure 2A shows the main window of the software that controls the robotic system.

c) Robot drivers

A DC supply (24 V, 6 A) is used to drive the Shinsei drivers. The control of the robot movement is achieved via a USB 6251 data acquisition (DAQ) interface card (National instruments, Austin, Texas, USA). Figure 2B shows the enclosure that hosts the electronic drivers of the robotic system. This enclosure contains magnetic materials, and therefore, it is placed outside the MRI room.

### HIFU system

The functionality of the robot was evaluated by creating discrete and overlapping thermal lesions using HIFU in gel phantoms (ONDA Corporation, Sunnyvale CA, USA). The HIFU system consists of a signal generator (HP 33120A, Agilent technologies, Englewood, CO, USA), an RF amplifier (250 W, AR, Souderton, PA, USA), and a spherical transducer made from piezoelectric ceramic (Etalon, Lebanon, IN, USA). The transducer operates at 3 MHz and has focal length of 4 cm and diameter of 3 cm.

### Experiments in gel phantoms

Experiments in gel phantom were carried out in order to evaluate the repeatability and functionality of the robotic device. The gel under evaluation was placed in a degassed water tank. The transducer was placed on the arm of the positioning device and was immersed in the water tank, thus providing good acoustical coupling between the gel and transducer.

### MR imaging

The 3D robotic HIFU system was initially tested in a 1.5-T MR system (Signa, General Electric, Fairfield, CT, USA) using a spinal coil (USA instruments, Cleveland, OH, USA) to acquire the MRI signal. In order to test the MRI compatibility of the positioning device, the DQA phantom (NiCl2, H20) by GE-Dielectric Corporation (Menomonee Falls, WI, USA) was used. The signal to noise ratio (SNR) was measured under various conditions (motor activation and encoder activation).

High-resolution MR imaging was performed to measure the SNR in the liquid phantom and to visualize HIFU lesions in a gel phantom. For imaging, a T2-weighted fast spin echo sequence was used with the following parameters: repetition time (TR): 2,500 ms, echo time (TE) = 60 ms, slice thickness = 3 mm (gap 0.3 mm), matrix = 256 × 256, field of view (FOV) = 16 cm, number of excitations (NEX) = 3, and echo train length (ETL) = 8.

For fast imaging that is used for MRI thermometry, T1-weighted fast spoiled gradient (FSPGR) was used with the following parameters: TR = 50 ms, TE = 2.7 ms, FOV = 16 cm, matrix = 256 × 256, flip angle = 30°, and NEX = 1.

## Results

**Figure 3:**
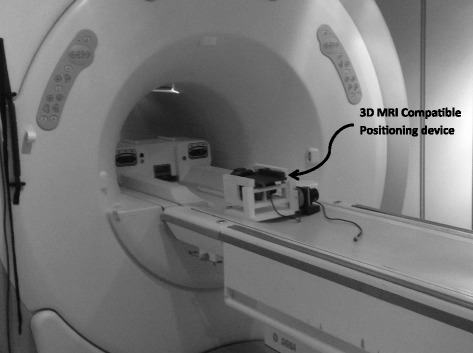
**Photo of the 3D positioning device as placed on the table of the GE MRI scanner.**

Figure 3 shows the positioning device placed on the table of the GE MRI scanner. Because of the size of the device (25 × 20 × 15 cm) fits potentially in any 1.5- or 3-T MRI scanner.

**Figure 4:**
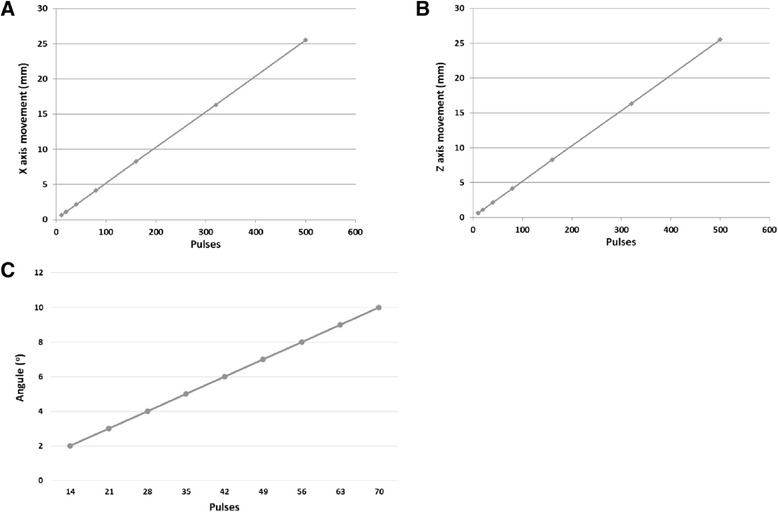
**Graphs of different accuracy tests. (A)** Measured distance vs. the number of cycles of the encoder for the forward linear motion for the *X*-axis, **(B)** measured distance vs. the number of cycles for the forward linear motion for the *Z*-axis, and **(C)** measured angle vs. the number of cycles of the encoder for the angular axis.

Figure 4A shows the graph of measured distance of the encoder vs. the number of cycles of the encoder for the forward linear motion for the *X*-axis. The relationship between the measured distance and number of pulses of the encoder pulses is linear. When the number of pulses is zero, the distance moved is not zero, because during the execution of the programming code in C, some overhead time is always needed. Therefore, as soon as the distance to be moved is chosen, the program extracts the number of pulses that the encoder has to measure. The constant slope from this graph depends on the encoder type (for the linear encoder is about 200 pulses/cm). During the evaluation test at any given *X*, 100 measurements were acquired. The average error measured for all steps was 23 μm (standard deviation of 6 μm). Similar results were obtained when the linear stage was moved in reverse direction (19 μm with standard deviation of 4.8 μm). The average error in the other linear stage (*Z*-axis) was 22 μm (standard deviation of 4.5 μm) for the upwards movement and 19 μm (standard deviation of 3.7 μm) for the downwards movement. The relationship between the number of pulses and the measured distance in the *Z*-axis is shown in Figure 4B. The error for the angular stage (theta axis) was 0.11° (standard deviation of 0.02°) for clockwise movement and 0.12° (standard deviation of 0.02°). The relationship between the number of pulses and the measured angle is shown in Figure 4C.

**Figure 5:**
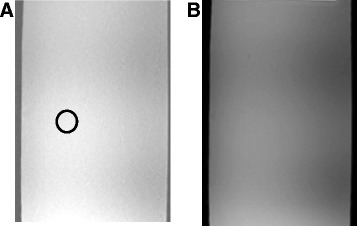
**T1-W FSPGR imaging of the gel phantom with and without activation of motor. (A)** T1-W FSPGR imaging of the gel phantom without activation of motor, encoder, and transducer and **(B)** T1-W FSPGR imaging of the gel phantom with activation of motor, encoder, and transducer.

Figure 5A shows the T1-weighted (W) FSPGR imaging of the gel phantom without activation of motor, encoder, and transducer, and Figure 5B shows the T1-W FSPGR imaging of the gel phantom with activation of motor, encoder, and transducer.

**Figure 6:**
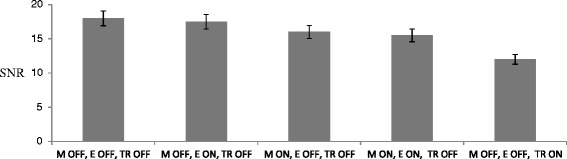
**Signal to noise ratio (SNR) measured for different conditions using T1-W FSPGR using the liquid phantom.**

Figure 6 shows the SNR measured for different device activations (piezoelectric motor, optical encoder, and transducer) of the liquid phantom using T1-weighted (W) FSPGR. The piezoelectric motor, optical encoder, and transducer require the use of electricity during activation. The SNR is maximum when the motor, encoder, and transducer are deactivated. There is some decrease in the SNR when an encoder is activated, and further decreased is observed when a motor is activated. More decrease in SNR is observed when the transducer is activated. The activation of the transducer causes the largest SNR drop. Obviously, the maximum drop of SNR occurs when the motor, encoder, and transducer are activated. The SNR drop with the worst case is significant but does to restrict the MRI imaging, since the motors and encoders are quite far from the imaging plane. Similar drop in the MRI signal due to the activation of piezoelectric motors has been reported also by [31].

**Figure 7:**
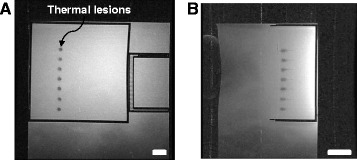
**Discrete thermal lesions created in the gel phantom.** Using the linear stage of the positioning device imaged using T2-W FSE in a 1.5-T GE scanner. The intensity used was 1,500 W/cm^2^ (spatial average *in situ*) for 10 s. The spacing between the lesions was 10 mm. The bar corresponds to 5 mm. **(A)** Perpendicular to the transducer beam and **(B)** parallel to the transducer beam. The bar corresponds to 10 mm.

Figure 7 shows thermal lesions in the gel phantom by moving the *X*-axis of the robotic system using discrete steps of 10 mm. The images were obtained with the 1.5-T GE MRI scanner using T2-weighted FSE. The ultrasound intensity used was 1,500 W/cm^2^ (spatial average *in situ*) for 10 s. These lesions were placed 3 cm deep in the gel. Figure 7A shows the lesion in a plane perpendicular to the transducer beam. The average diameter of the lesions is 2.4 mm (standard deviation is 0.2 mm). Note that the spacing between the center of lesions is consistently 10 mm (measured using MRI imaging) which demonstrates the excellent repeatability of the robotic system. The length of the lesions parallel to the focal beam as shown in Figure 7B is around 17 mm.

**Figure 8:**
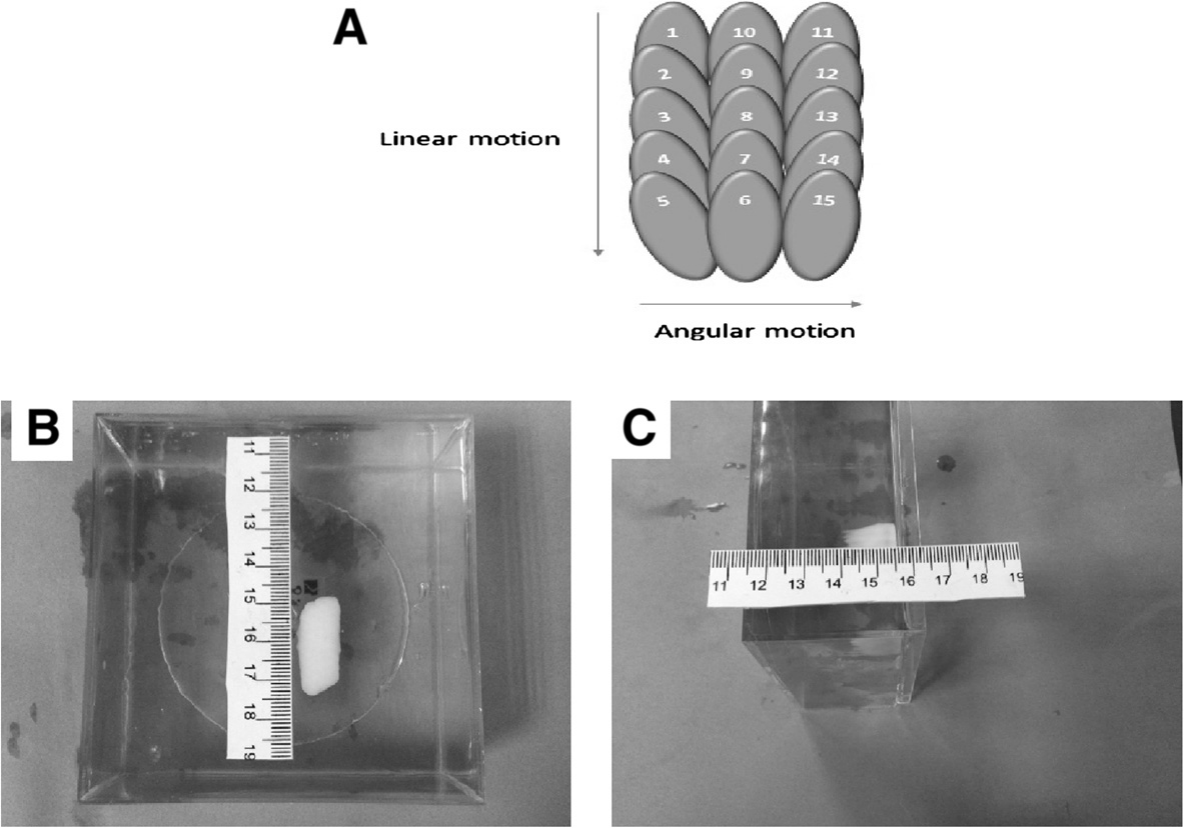
**Strategy of creating overlapping lesions and thermal lesions and the grid ablation from a plane. (A)** Strategy of creating overlapping lesions by moving the linear axis *X* and the angular axis *θ* (example of five linear steps and three angular steps). **(B)** Overlapping thermal lesions created in gel phantom using both the linear and angular stages of the positioning device. The intensity used was 1,200 W/cm^2^ (spatial average *in situ*) for 10 s. The ablation grid was 10 × 5 with spacing between lesions of 2 mm for the linear stage and 3° for the angular stage. The plane is perpendicular to the ultrasonic beam. **(C)** The plane is parallel to the ultrasonic beam.

Figure 8A shows the strategy of creating overlapping lesions by moving the linear axis *X* and the angular axis *θ* (example of five linear steps and three angular steps). Figure 8B shows overlapping thermal lesions created in the gel phantom by moving the transducer in the *X*-axis and in the *Θ*-axis. The intensity used was 1,200 W/cm^2^ (spatial average *in situ*) for 10 s. The ablation grid was 10 × 5 (i.e., ten linear steps and five angular steps). The spacing between lesions was 2 mm for the *X*-axis and 3° for the *Θ*-axis. Figure 8B shows the grid ablation from a plane perpendicular to the transducer beam. Because the lesions were created in a sequential pattern linearly and angularly, the shape of the large lesion was affected by near-field heating and as a result, the shape shown in this plane is not rectangular as expected. Figure 8C shows the grid ablation from a plane parallel to the transducer beam showing that the depths of the lesions extend to approximately 15 mm. This overlapping large lesion was created in the surface, and therefore, less intensity was needed compared to the lesions of Figure 7.

## Discussion

Based on the location of the prostate, the existing HIFU systems [3,4,13-16] cannot be used because the penetration of ultrasound to the prostate gland will be poor. Therefore, a transrectal FUS system navigated by a robotic system is needed.

Currently, another MRI-guided HIFU system exists for prostate interventions using a transurethra approach [22]. In the transurethra system, the transducer which is unfocused delivers heat that moves from the probe to the targeted area. Therefore, tissue that lies between the transducer and the target is unnecessarily heated. In our system, the transducer is focused, and therefore, heat can be delivered only to the target. In the transrectal approach, there is more space and therefore, the transducer can be focused, as opposed to the transurethra approach where the transducer has to be unfocused due to the limited space in the urethra. This focusing capability of the transrectal system could be beneficial for focal therapy. On the other hand, the transurethra has the advantage of being a much simpler technology.

The enclosure that accommodates the transducer of the proposed system is inserted through the rectum. Due to the variable height of the prostate from the patient table, there is a need to lift the probe automatically. Therefore, the proposed system includes a linear stage for lifting up or down the transducer enclosure. The proposed positioning device assumes a straight access to the rectum, but in reality, an angular offset exists. Therefore, in the future, a modification needs to be done in the system so that the *X*-axis could move at an angle.

Regarding the functionality of the HIFU transducer, a linear and an angular axis is needed in order to ablate large volume of prostate tissue. We have showed that it is possible to construct a robotic system that can move the transducer accurately, thus creating large thermal lesions. This has been proven in experiments conducted in commercial gel phantoms.

During the tests in the MR system, some degradation of the image quality was observed. The SNR drops when the piezoelectric motor or the optical encoder or the transducer is activated. Most of the drop in the SNR was attributed to the transducer which requires more energy during activation and also due to its close proximity to the imaging plane. Nevertheless, the drop of the SNR due to the activation of the piezoelectric motors and optical encoders did not significantly alter the MRI imaging, since these two components are placed few centimeters away from the imaging coil. Most of the drop in the SNR is caused by the transducer which is placed in closer proximity to the imaging plane.

The measurement error for the linear axes is around 20 μm and for the angular axis is around 0.11°. The accuracy of the movement of the stages was assessed by measuring the actual distance moved compared to the intended distance. The accuracy was also demonstrated in a realistic scenario by accurately creating lesions in gel phantoms.

More extensive evaluation will be needed in the future in animal experiments, which will be done most likely in dogs (as in the study by [20]) due to the similar position and size of the prostate in the rectum. The success of HIFU in treating prostate tissue under ultrasonic imaging has been already established [19]. A technology using HIFU and MRI guidance as proposed here has to be evaluated extensively in animal experiments. This could prove the added benefit of using MRI guidance, since MRI can image thermal lesions with excellent contrast [32]. Another critical issue is the selection of a proper imaging coil which is currently under construction. The coil will be incorporated in the water container of the transducer which makes tuning and matching of this coil more problematic [33].

The proposed robot as it is currently designed hosts a single element transducer. In the future, a better but more expensive choice is to use a phased array. The phased array technology has the added functionality that the depth of the focal beam can be varied electronically with the cost of additional cabling. However, there will be a limit in the number of elements that can be used due to space constrains in the transducer enclosure. The proposed system can be easily altered in order to treat gynecological tumors endovaginally. This application can be achieved by modifying the transducer holder (smaller dimensions are allowed for gynecological applications).

The proposed device has been proven to be very accurate, and therefore, the ultimate goal is to utilize it in the clinical environment, provided that experience is gained during preclinical trials in animals.

## References

[1] Hynynen K, Damianou C, Darkazanli A, Unger E, Schenck J (1992). The feasibility of using MRI to monitor and guide noninvasive ultrasound surgery. Ultrasound Med Biol.

[2] Cline H, Rohling K, Abeling W (1995). Mechanical positioner for magnetic resonance guided ultrasound therapy.

[3] Yehezkeli O, Freundlich D, Magen N, Marantz C, Medan Y, Vitek S, Weinreb A. Mechanical positioner for MRI guided ultrasound therapy system. Inventors word intellectual property organization, INSIGHTEC-TXSONICSLTD, assignee, WO0209812. 2002.

[4] Stewart A, Gedroyc W, Tempany M, Quade B, Inbar Y, Ehrenstein T (2003). Focused ultrasound treatment of uterine fibroids: safety and feasibility of a noninvasive thermoablative technique. Am J Obstet Gynecol.

[5] Tempany M, Stewart A, McDannold N, Quade B, Jolesz F, Hynynen K (2003). MRI guided focused ultrasound surgery (FUS) of uterine leiomyomas: a feasibility study. Radiology.

[6] Stewart EA, Rabinovici J, Tempany C, Inbar Y, Regan L, Gostout B (2006). Clinical outcomes of focused ultrasound surgery for the treatment of uterine fibroids. Fertil Steril.

[7] Kim HS, Baik JH, Pham LD, Jacobs MA (2011). MR-guided high-intensity focused ultrasound treatment for symptomatic uterine leiomyomata long-term outcomes. Acad Radiol.

[8] Funaki K, Fukunishi H, Funaki T, Kawakami C (2007). Mid-term outcome of magnetic resonance-guided focused ultrasound surgery for uterine myomas: from six to twelve months after volume reduction. J Minim Invasive Gynecol.

[9] Laveena P, Vinay N, Anindita M, Himabindu Y, Mythri V (2012). Noninvasive treatment of focal adenomyosis with MR-guided focused ultrasound in two patients. Indian J Radiol Imaging.

[10] Zini C, Elisabeth H, Stephen T, Alessandro N, Carlo C, Aytekin O (2012). Ultrasound- and MR-guided focused ultrasound surgery for prostate cancer. World J Radiol.

[11] Napoli A, Anzidei M, De Nunzio C, Cartocci G, Panebianco V, Dominicis C, Catalano C, Petrucci F, Leonardo C (2013). Real-time Magnetic Resonance–guided High-intensity Focused Ultrasound Focal Therapy for Localised Prostate Cancer: Preliminary Experience. Eur Urol.

[12] Napoli A, Anzidei M, Marincola C, Brachetti G, Ciolina F, Cartocci G (2013). Primary Pain Palliation and Local Tumor Control in Bone Metastases Treated With Magnetic Resonance-Guided Focused Ultrasound. Investig Radiol.

[13] Lee E, Yoon W, Kim A, Lee T, Shay L, Lee S (2011). Successful use of magnetic resonance-guided focused ultrasound surgery for long-term pain palliation in a patient suffering from metastatic bone tumor. Korean Soc of Radiol.

[14] Catane R, Beck A, Inbar Y, Rabin T, Shabshin N, Hengst S (2006). MR-guided focused ultrasound surgery (MRgFUS) for the palliation of pain in patients with bone metastases—preliminary clinical experience. Ann Oncol.

[15] McDannold N, Clement G, Black P, Jolesz F, Hynynen K (2010). Transcranial MRI-guided focused ultrasound surgery of brain tumors: **I**initial findings in three patients. Neurosurgery.

[16] Dorenberg J, Courivaud F, Ring E, Hald K, Jakobsen Å, Fosse E (2013). Volumetric ablation of uterine fibroids using Sonalleve high-intensity focused ultrasound in a 3 Tesla scanner—first clinical assessment. Minim Invasive Therapy Allied Technol.

[17] Bihrle R, Foster S, Sanghvi T, Frank F, Donohue J (1994). High-intensity focused ultrasound in the treatment of prostatic tissue. Urology.

[18] Gelet A, Chapelon Y, Margonari J, Theillère Y, Gorry F, Souchon R (1993). High intensity focused ultrasound experimentation on human benign prostatic hypertrophy. Eurpean Urol.

[19] Chaussy C, Thuroff S (2003). The status of high-intensity focused ultrasound in the treatment of localized prostate cancer and the impact of a combined resection. Urol Rep.

[20] Madersbacher S, Marberger M (2003). High-energy shockwaves and extracorporeal high-intensity focused ultrasound. J Endourol.

[21] Saleh Y, Smith B (2005). A 63 element 1.75 dimensional ultrasound phased array for the treatment of benign prostatic hyperplasia. Adv Biomed Eng Med Phys.

[22] Chopra R, Baker N, Choy V, Boyes A, Tang K, Bradwell D (2008). MRI-compatible transurethral ultrasound system for the treatment of localized prostate cancer using rotational control. Med Phys.

[23] Chopra R, Tang K, Burtnyk M, Boyes A, Sugar L, Appu S (2009). Analysis of the spatial and temporal accuracy of heating in the prostate gland using transurethral ultrasound therapy and active MR temperature feedback. Physics Medical Biol.

[24] Stoianovici D, Song D, Petrisor D, Ursu D, Mazilu D, Mutener M (2007). MRI stealth robot for prostate interventions. Minim Invasive Therapy Allied Technol.

[25] Zangos S, Herzog C, Eichler K, Hammerstingl R, Lukoschek A, Guthmann S, Gutmann B, Schoepf J, Costello P, Vogl T (2007). MR-compatible assistance system for punction in a high-field system: device and feasibility of transgluteal biopsies of the prostate gland. Eur Urol.

[26] Shan J, Jie G, Shen L, Jun L, Jun Y (2012). Kinematic analysis of a 5-DOF hybrid-driven MR compatible robot for minimally invasive prostatic interventions. Robotica.

[27] Mylonas N, Damianou C (2013). MR compatible positioning device for guiding a focused ultrasound system for the treatment of brain diseases. Int J Med Robotics Computer Assisted Surgery.

[28] Damianou C, Ioannides K, Hadjisavas V, Milonas N, Couppis A, Iosif D, Kyriacou P (2010). MRI monitoring of lesions created at temperature below the boiling point and of lesions created above the boiling point using high intensity focused ultrasound. J Biomedical Science and Engineering.

[29] Hadjisavvas V, Ioannides K, Komodromos M, Mylonas N, Damianou C (2010). Evaluation of the contrast between tissues and thermal lesions in rabbit in vivo produced by high intensity focused ultrasound using fast spin echo MRI sequences. J Biomedical Science and Engineering.

[30] Stoianovici D, Chunwoo K, Srimathveeravalli G, Sebrecht P, Petrisor D, Coleman J (2014). MRI-safe robot for endorectal prostate biopsy. Mechatronics.

[31] Fischer G, Krieger A, Iordachita I, Csoma C, Whitcomb L, Fichtinger G (2008). MRI compatibility of robot actuation techniques—a comparative study. Computer-Assisted InterventionMed Image Comput Comput Assist Interv.

[32] Mylonas N, Ioannides K, Hadjisavvas V, Iosif D, Kyriacou P, Damianou C (2010). Evaluation of fast spin echo MRI sequence for an MRI guided high intensity focused ultrasound system for in vivo rabbit liver ablation. J Biomedical Sci and Engineering.

[33] Pavlina M, Groebner J, Dadakova T, Dumont E, Bock M. Design of an endorectal coil for MR-guided HIFU therapy of the prostate. Proc. Intl. Soc. Mag. Reson. Med., 2013, 1797.

